# Surgical timing prevails as the main factor over morphologic characteristics in the reduction by ligamentotaxis of thoracolumbar burst fractures

**DOI:** 10.1186/s12893-023-02061-z

**Published:** 2023-06-20

**Authors:** Juan Ignacio Cirillo, Ignacio Farias, Cristóbal Del Pino, Marcos Gimbernat, Alejandro Urzúa, Carlos Tapia, Juan José Zamorano

**Affiliations:** 1 Hospital del Trabajador, Santiago, Chile; 2 Clínica Universidad de los Andes, Santiago, Chile; 3 Hospital San José, Santiago, Chile; 4 Clínica Dávila Vespucio, Santiago, Chile; 5 Universidad Andrés Bello, Hospital del Trabajador, Facultad de Medicina, Santiago, Chile 8370035 República 239,; 6 Instituto Traumatológico, Santiago, Chile; 7 Clínica Alemana, Santiago, Chile; 8 Universidad del Desarrollo, Santiago, Chile

**Keywords:** Thoracolumbar, Burst fracture, Decompression, Trauma, CT, Spinal cord injury, Fixation

## Abstract

**Background:**

thoracolumbar burst fractures are associated with spinal canal occupation. The indirect decompression of the spinal canal and reduction of the fragment can be achieved with the distraction of the middle column and ligamentotaxis. Nevertheless, the factors that influence the effectiveness of this procedure and its temporality are controversial.

**Methods:**

The aim of this observational, cross-sectional study was to evaluate the effectiveness of indirect reduction by ligamentotaxis in thoracolumbar burst fractures according to the fracture’s radiologic characteristics and the procedure’s temporality. Patients diagnosed with a thoracolumbar burst fracture between 2010 and 2021 were submitted to indirect reduction by distraction and ligamentotaxis. A retrospective analysis of radiologic characteristics and temporality of the procedure was performed with an independent sample t-test or Pearson’s correlation coefficient, as required.

**Results:**

A total of 58 patients were included in the analysis. Postoperatively, ligamentotaxis significantly improved all radiologic parameters (canal occupation, endplates distance, and vertebra height). Still, none of the radiological characteristics of the fracture (width, height, position, sagittal angle) were associated with the postoperative change in canal occupation. The endplates distance and the temporality of ligamentotaxis significantly predicted the reduction of the fracture.

**Conclusion:**

Fragment reduction effectiveness is more significant when performed as early as possible and adequate distraction is achieved using the internal fixator system. The radiologic characteristics of the fractured fragment do not determine its reduction capacity.

## Background

Thoracolumbar burst fractures are characterized by the comminuted compromise of the middle Denis column and are typically associated with some degree of medullary canal occupation [1].

Both the indirect decompression of the medullary canal and the fragment reduction can be achieved by the distraction of the middle column by ligamentotaxis, the procedure by which the retropulsed fragment can be reduced using the posterior longitudinal ligament (PLL) and the annulus fibrosus as a tension band.

Some studies suggest that the fragment reduction capacity is influenced by the morphology of the lesion, but their results are divergent from each other, and there is no association between the temporality of the procedure and the fragment reduction capacity [2–9].

Therefore, the purpose of this study was to evaluate the effectiveness of indirect reduction by ligamentotaxis in thoracolumbar burst fractures according to the fracture’s radiologic characteristics and the procedure’s temporality.

## Methods

### Study design

An observational, cross-sectional study was conducted in a level I trauma center between 2010 and 2021, including patients over 18 years old presenting with a thoracolumbar burst fracture with a retropulsed fragment submitted to indirect reduction by distraction and ligamentotaxis. The primary purpose of this study was to evaluate the effectiveness of indirect reduction by ligamentotaxis in thoracolumbar burst fractures according to the fracture’s radiologic characteristics and the procedure’s temporality.

Patients with thoracolumbar fractures type B or C (as defined by the AO Spine Classification), pathological bone fractures, ankylosing spondylitis, or diffuse idiopathic skeletal hyperostosis (DISH) were excluded from the study. Patients without sufficient imaging to compare preoperative and postoperative radiological changes on CT scans were also excluded. Additionally, patients with a Reverse or Pseudoreverse Cortical Sign (as described by Arlet and Cols. [9]) and those who required direct impaction after ligamentotaxis were excluded.

The study was approved by the local institutional ethics committee (CEC HT-24/2020), and informed consent was obtained from all participants. To protect patient privacy, a non-sequential coding system was used for the registry.

### Procedure protocol

Patients were placed in the prone position, and surgery was performed under general anesthesia. Schanz screws were used as internal fixators (USS fracture system, J&J). Ligamentotaxis by distraction was performed for the reduction of the retropulsed fragment [2–4].

### Variables and radiological measurements

Demographic data, such as age, sex, comorbidities, and tobacco use, along with characteristics of the primary injury, such as injury mechanism, segment involvement, multi-segment involvement, AO Spine Classification, and neurological impairment, were recorded to characterize the sample and to identify the possible factors that may also influence the effectiveness of the procedure. The temporality of the reduction by ligamentotaxis was recorded as the number of days between the primary injury and the definitive surgery.

Radiologic measurements were made on two-dimensional CT scans at the time of admission, and professional radiologists of our institution carried out the first postoperative imaging control.

**Fig. 1:**
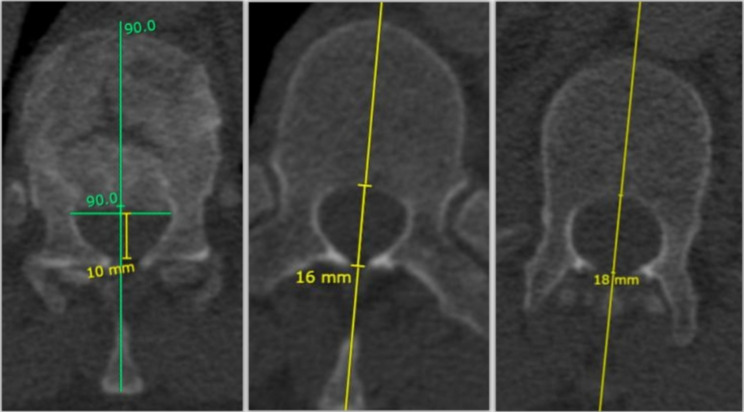
From **left** to **right**: distance between the retropulsed fragment and the posterior end of the canal; anteroposterior (AP) diameter of the medullary canal of the above vertebrae; anteroposterior (AP) diameter of the medullary canal of the below vertebrae. The medullary canal occupation was calculated as the subtraction of the first measurement and the mean AP diameter of the adjacent segments

**Fig. 2:**
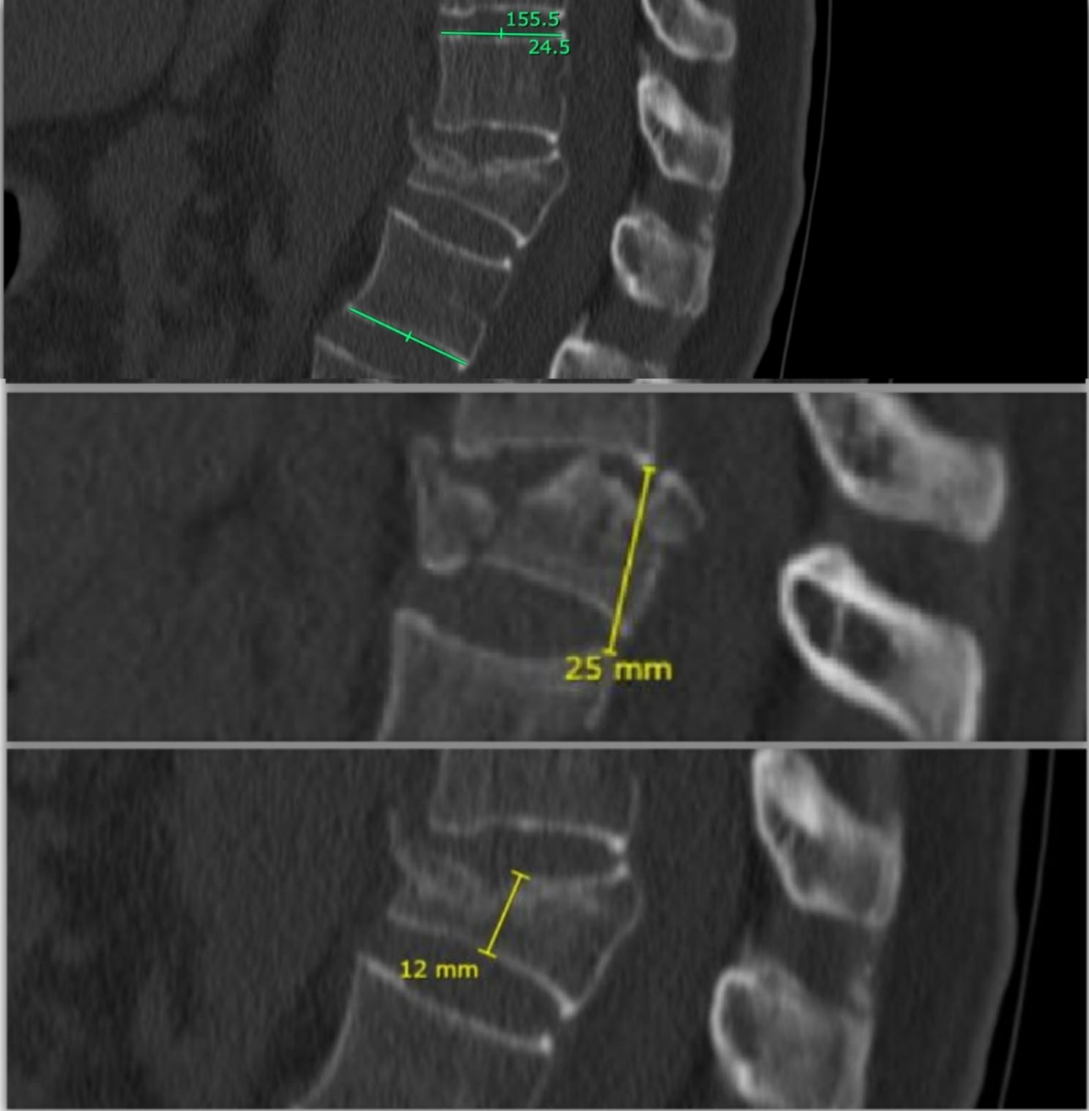
**Above**: bisegmental kyphosis. **Middle**: endplate distance. **Below**: vertebrae height. **Green** lines: angle measures. **Yellow** lines: distance measures

**Fig. 3:**
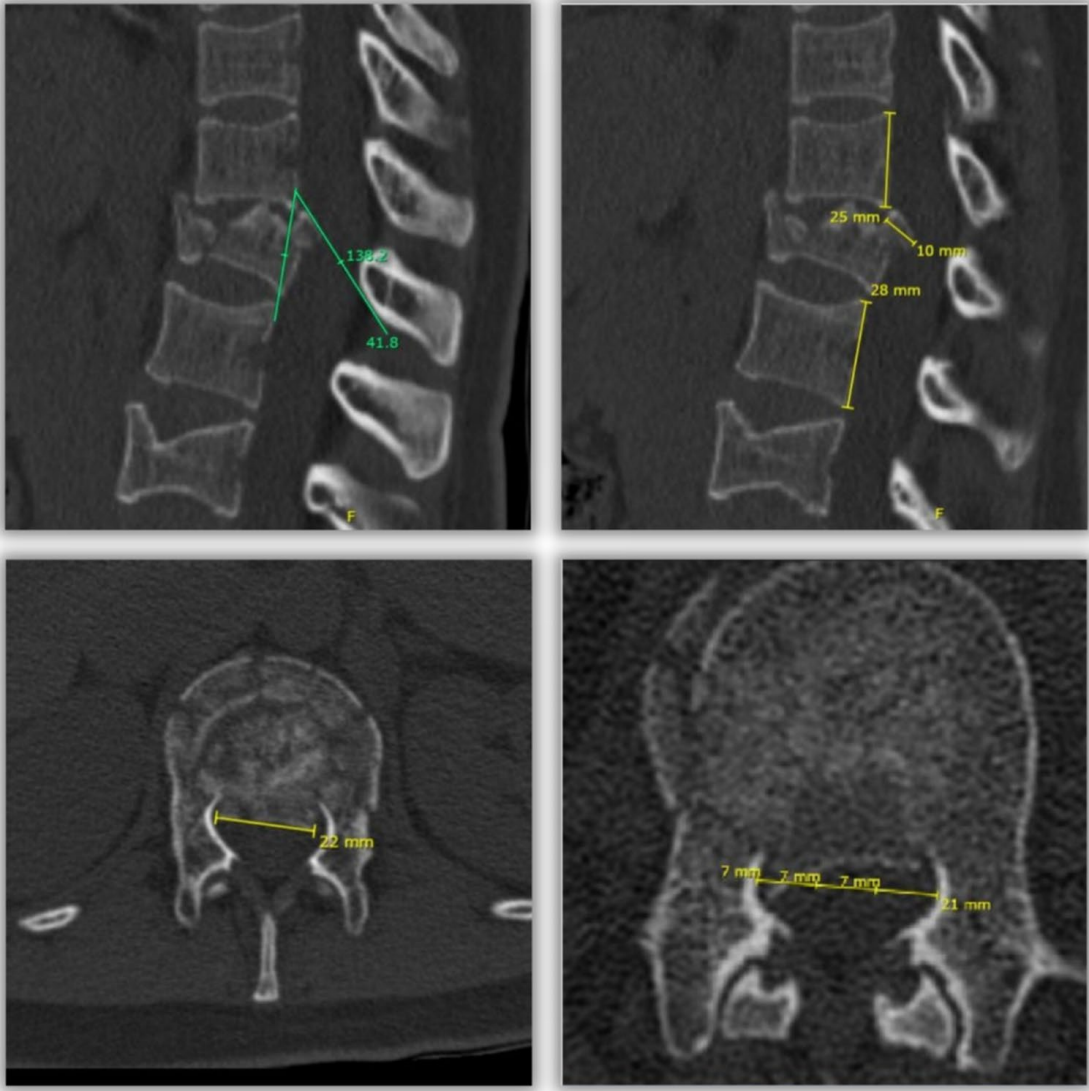
**Upper left image**: sagittal angle. **Upper right image**: fragment height. **Lower left image**: fragment width. **Lower right image**: position of the retropulsed fragment in the medullary canal –in this case the fragment was lateralized–. **Green lines**: angle measures. **Yellow lines**: distance measures

These measurements were as follows:

- **Medullary canal occupation** (%): this measure was performed on axial reconstruction by drawing a transverse line to the posterior tip of the retropulsed fragment and then obtaining the perpendicular distance between this line and the posterior end of the canal. The canal occupation was the subtraction of this measure from the mean anteroposterior (AP) diameter of the medullary canal of the adjacent segments, expressed as a percentage of the mean AP diameter of the adjacent segments. See Fig. 1.

- **Endplates distance** (mm): the height in millimeters between the posterior vertex of the superior endplates of the segment below the injury and the posterior vertex of the inferior endplate of the segment above the injury, measured on sagittal reconstruction at the middle of the spine in the coronal plane. See Fig. 2.
- **Vertebra height** (mm): the height in millimeters of the middle body vertebra of the injured segment measured on sagittal reconstruction at the middle of the spine in the coronal plane. See Fig. 2.
- **Bisegmental kyphosis** (°): the angular degrees of the inferior endplate of the segment below and the superior endplates of the segment above on sagittal reconstruction. See Fig. 2.

- **Sagittal angle** (°): the angular degrees of the posterior wall of the retropulsed fragment compared to a straight line connecting the posterior walls of the adjacent segments measured on sagittal reconstruction, where the posterior wall of the retropulsed fragment was the longest. See Fig. 3.
- **Fragment width** (mm): measured in millimeters on axial reconstruction at the fragment’s widest level. This parameter was presented as a percentage of this measure compared to the mean transverse diameter of the adjacent segments. See Fig. 3.
- **Fragment height** (mm): measured in millimeters on sagittal reconstruction at the level where the fragment had the longest posterior wall by drawing a perpendicular line connecting the articular surface and the opposite tip of the retropulsed fragment. This parameter was presented as a percentage of this measure compared to the mean posterior wall distance of the adjacent segments. See Fig. 3.
- **Position of the retropulsed fragment in the medullary canal**: the position of the retropulsed fragment in relation to the middle third (central) or lateral thirds (lateral) of the medullary canal on axial reconstruction where the canal was occupied the most. See Fig. 3.

### Statistical analysis

Descriptive statistics were performed using the mean and standard deviation, or the median and interquartile range (IQ_25 − 75_), as needed, for each variable. Postoperative changes in the radiological parameters were evaluated with a paired sample t-test. The association of the changes in spinal canal occupation with the radiologic characteristics and the temporality of the procedure was assessed with an independent sample t-test or Pearson’s correlation coefficient, as required. A multiple regression analysis was also performed in a stepwise manner, including demographics and injury characteristics, and radiological parameters were evaluated. Results were calculated using STATA BE v17.0. The imaging studies were evaluated using the Agfa Xero Viewer ver. 8.1.2 (2017) software (Agfa HealthCare, Mortsel, Belgium).

## Results

### Patient characteristics

There were fifty-eight patients included in the analysis (see Table 1). The mean age was 47.10 ± 13.68, and the sex ratio 5:2 (M:F). The most frequent segments involved were L1 (51.72%), T12 (24.14%), and L2 (13.79%), and the median temporality of the procedure was 3 [2, 7] days.

**Table 1:** Sample characteristics

Age (years)	47.10 ± 13.68
Sex (M:F)	5:2
Comorbidities Arterial Hypertension Diabetes Mellitus Coronary disease Hypothyroidism Other	19(32.76%) 10 (17.24%) 7 (12.07) 1 (1.72%) 2 (3.45%) 5 (8.62%)
Smoking	19 (32.76%)
Mechanism	
Fall from height Automovilistic accident Forced sitting Crush injury Level ground fall Bicycle accident	24 (41.38%) 14 (24.14%) 8 (13.79%) 5 (8.62%) 5 (8.62%) 2 (3.45%)
Temporality of ligamentotaxis (days)	3 [2;7]*
Segment involved	
T12 L1 L2 L3 L4 L5	14 (24.14%) 30 (51.72%) 8 (13.79%) 2 (3.45%) 3 (5.17%) 1 (1.72%)
AOspine Classification (A4:A3)	5:2
Neurologic impairment	8 (14.55%)
Multilevel fractures (≥ 2 segments)	10 (17.24%)
**Total**	58

*Interquartile range

### Surgery-related variables

Postoperatively, ligamentotaxis significantly improved all radiologic parameters included in this study (see Table 2). Most fragments were retropulsed at the central third of the medullary canal instead of lateralized, at a proportion of 4:1; however, this was not associated with the percentage of canal occupation change after ligamentotaxis (15.89% vs. 12.56%, p = 0.380). Moreover, none of the radiological parameters or fragment dimensions were associated with the postoperative change in canal occupation (see Table 2).

**Table 2:** Radiologic characteristics of the fracture

	Preoperative	Postoperative	∆	p-value*
Canal occupation (%)	42.66 ± 13.81	28.80 ± 15.55	13.87 ± 13.16 IC 10.38–17.36	p < 0.0001
Endplates distance (mm)	33.33 ± 3.94	36.79 ± 4.62	3.47 ± 3.65 IC 2.51–4.43	p < 0.0001
Vertebra height (%)	67.19 ± 12.02	78.28 ± 15.23	11.09 ± 16.14 IC 6.85–15.33	p < 0.0001
Sagittal angle (º)	24.01 ± 15.75	16.40 ± 12.40	7.61 ± 12.50 IC 4.29–10.93	p < 0.0001
Bisegmental kyphosis (º)	7.02 ± 13.49	1.26 ± 11.93	5.76 ± 6.22 IC 4.12–7.40	p < 0.0001
Width (%)	74.46 ± 17.58			
Height (%)	47.15 ± 8.32			
Position (C:L)ˤ	4:1			

*Paired sample t-test comparing pre- and postoperative change of each parameter

ˤCentral (C) or lateral (L) intracanal position of the retropulsed fragment in the axial plane

**Fig. 4:**
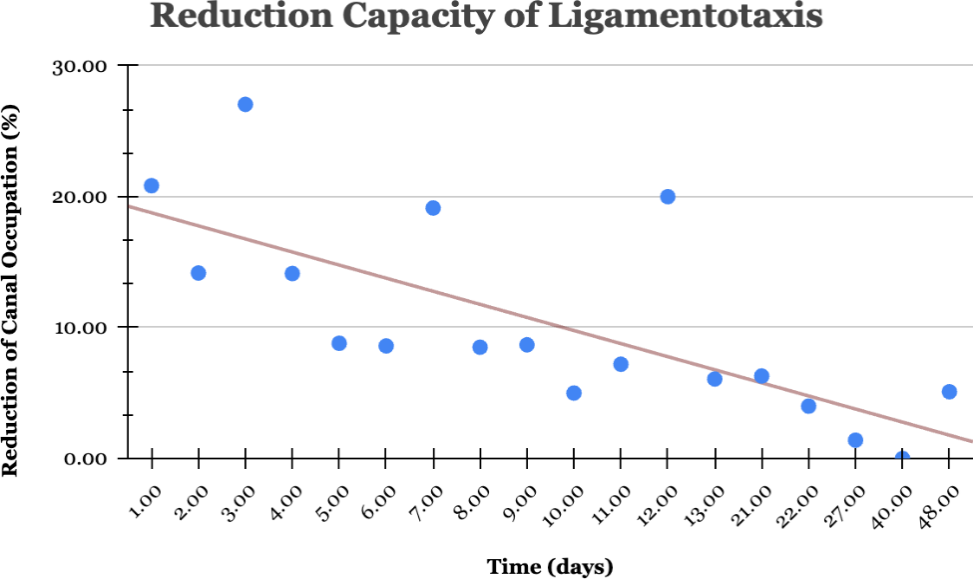
Ligamentotaxis capacity of reduction by its temporality. The **blue dots** show the mean change in canal occupation for every day of delay in the procedure. The **red line** represents the tendency line of this association

**Fig. 5:**
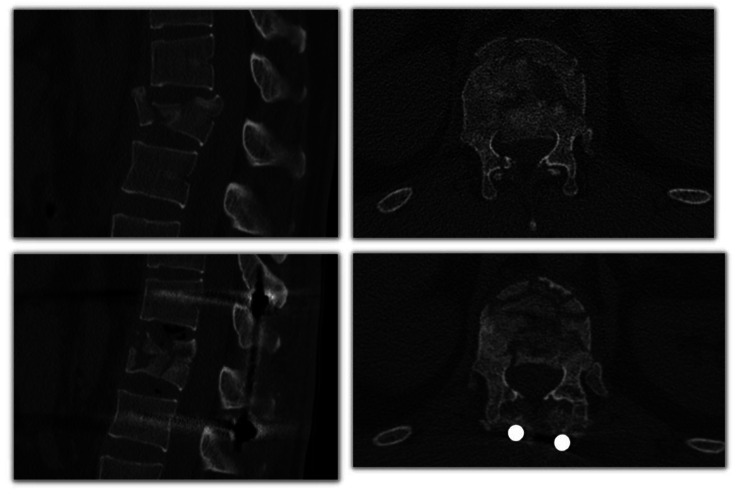
Case #1: ligamentotaxis performed in < 24 h. Canal occupation was reduced by 35%. **Above**: before ligamentotaxis; **Below**: after ligamentotaxis and fixation

**Fig. 6:**
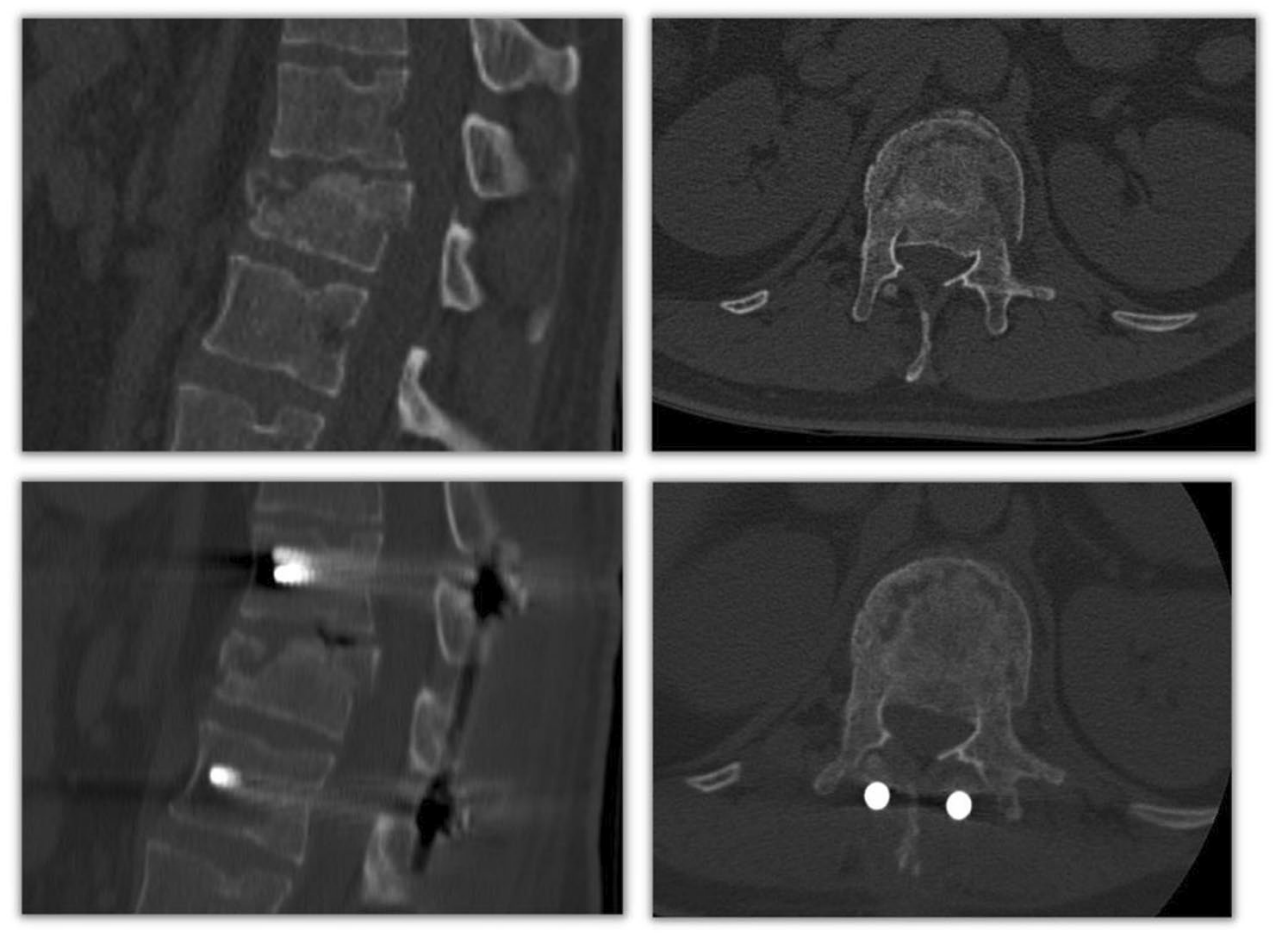
Case #2: ligamentotaxis performed 1 week after the accident. Canal occupation was reduced by 17%. **Above**: before ligamentotaxis; **Below**: after ligamentotaxis and fixation

**Fig. 7:**
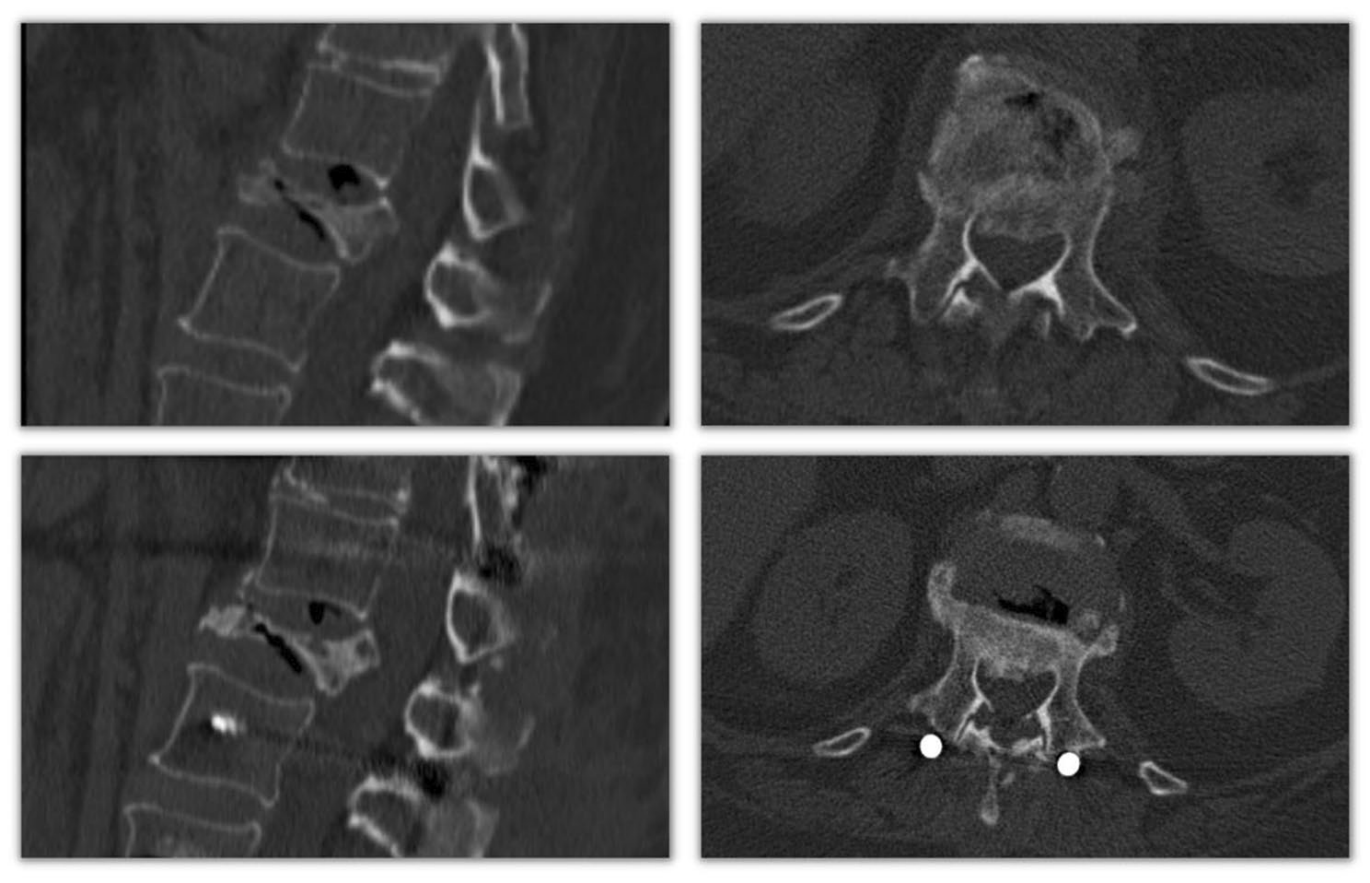
Case #3: ligamentotaxis performed 1 month after the accident. Canal occupation was reduced by 1.4%. **Above**: before ligamentotaxis; **Below**: after ligamentotaxis and fixation

Only the temporality of the procedure was associated with the procedure’s capacity for reduction (r = − 0.382); see Fig. 4. This means that the temporality of the procedure itself explained 14.59% (R2 = 0.146) of the canal occupation variability. For better understanding, examples are shown in Figs. 5, 6 and 7.

Nevertheless, when multiple regression analysis was performed, the endplates distance was also statistically significantly associated with this outcome through the following formula:

(Δ **Canal occupation**) = -0.57 ⋅ (**Temporality**)-0.92 ⋅ (**Endplates distance**) + 49.21.

[F (2,54) = 8.38, p = 0.0007, R2 = 0.236]

This means that the capacity of ligamentotaxis reduction was reduced by 2.85% every five days of delay in the procedure or 2.30% for every two millimeters less of endplate distance. This model explains 23.6% of the change in canal occupation variability.

## Discussion

Today, there is limited evidence available in the literature that compares the capacity for reduction by ligamentotaxis and if the morphology of the lesion and the timing of the surgery influence this [7–9].

Our results demonstrated that ligamentotaxis is effective for indirect reduction of thoracolumbar burst fractures with a retropulsed fragment. The capacity of reduction is associated with the temporality of the procedure and the endplates distance and not by the characteristics of the fragment itself.

Hu et al. studied the relationship between the size of the fragment and the disruption of the PLL on MRIs and found that the height and width of the fragment were statistically larger in those patients with a disrupted PLL [10]. This would suggest that the indirect reduction by ligamentotaxis alone may be insufficient in larger fragments because of the loss of the tension band when the PLL is disrupted. They also reported an association between the height of the fragment and the ability to reposition it, with an odds ratio of 5.2. We failed to reproduce these results in our study. Even though the association with the PLL status was not directly addressed, we did not find a correlation between the height or width of the fragment and the decompression of the canal occupation after ligamentotaxis. Even though it seems reasonable that the effectiveness of ligamentotaxis depends on the status of the PLL, if larger fragments do not relate to a lower reduction of the canal occupation, other factors may be involved in the effectiveness of ligamentotaxis besides the status of the PLL.

This issue has been addressed by other authors as well. In 2019, Chen et al. found a difference in the “mid-sagittal diameter compression ratio” (MSDCR) and the sagittal angle of the fragment in patients with a disrupted PLL compared to those with an intact PLL. They also found that the MSDCR and the sagittal angle could predict the disruption of the PLL when logistic regression was performed and established that when the MSDCR was 52%, and the sagittal angle was 33°, the PLL was likely to be disrupted [11]. Even so, they did not find a relationship between the size of the fragment and the disruption of the PLL, which indirectly correlates with our results.

The reducing capacity of ligamentotaxis is associated with the distance between the adjacent endplates and the timing of the procedure and not by the radiological characteristics of the fracture and its fragment. However, when analyzing the multiple regression, only 24% of the variability in the change in channel occupancy is explained by this formula. Therefore, it is valid to presume that other factors explain the rest of the channel occupancy variability that are not considered in it. It seems reasonable to think that the morphological characteristics of the fragment should influence its reduction capacity, but only a small number of authors have studied this relationship directly. In 2015, Dai et al. reported in a cross-sectional study of 55 patients a statistically significant inverse relationship between the “mid-sagittal canal diameter compression ratio”, measured as the ratio between the anteroposterior (AP) diameter of the injured segment canal and the mean AP diameter of the adjacent segments, and the capacity of repositioning the retropulsed fragment, which was also associated to the AO Spine Classification [7]. Even so, the authors did not find an association with the vertebrae height, bisegmental kyphosis, or the AP and transverse diameter of the fragment.

Also, in 2015, Peng et al. decided to study the association between the morphologic characteristics of the thoracolumbar fractures and the capacity of reduction by distraction and ligamentotaxis only in those patients presenting with a retropulsed fragment at the mid-third of the posterior vertebrae wall on the axial plane arguing that, from an anatomopathological point of view, the width of the PLL cannot reach the lateral thirds of the medullary canal and, therefore, the procedure would not be effective in this kind of patients. In this cross-sectional study of 55 patients, the authors found that the ratio of AP occupation of the medullary canal and the height and width of the retropulsed fragment were statistically significantly associated with the capacity of reduction of the procedure [8]. Both studies indicate that the amount of occupancy in the canal before the procedure is important for reduction. They also found that the AO spine classification and the dimensions of the fragment were associated with it. However, comparing our results is difficult because they used “reduction” as a dichotomic variable, without a clear definition. Furthermore, literature has yet to provide a clear definition of “reduction”. We defined reduction as a continuous variable, measuring the change in canal occupancy. Therefore, we cannot provide a recommendation for when to perform the procedure based on our results. Nevertheless, we suggest performing reduction by ligamentotaxis as soon as possible, especially within three weeks of the fracture, to prevent callus hardening.

## Conclusion

Further evidence is required to determine the optimal timing for surgery when performing reduction by ligamentotaxis. Our study focused solely on the percentage of occupation of the medullary canal as the reduction parameter. Interestingly, the radiologic characteristics of the fracture fragment did not impact its reduction capacity. Our findings demonstrate that a prompt reduction and effective distraction utilizing the internal fixator system can significantly enhance the percentage of fragment reduction.

## Data Availability

The datasets used and/or analyzed during the current study are available from the corresponding author on reasonable request.

## Abbreviations

PLL: Posterior longitudinal ligament
DISH: Idiopathic skeletal hyperostosis
CT: Computed tomography
AP: Anteroposterior
mm: Millimeters
IQ_25-75_: Interquartile range
MSDCR: Mid-sagittal diameter compression ratio
